# Upregulated expression of brain enzymatic markers of arachidonic and docosahexaenoic acid metabolism in a rat model of the metabolic syndrome

**DOI:** 10.1186/1471-2202-13-131

**Published:** 2012-10-30

**Authors:** Ameer Y Taha, Fei Gao, Epolia Ramadan, Yewon Cheon, Stanley I Rapoport, Hyung-Wook Kim

**Affiliations:** 1 Brain Physiology and Metabolism Section, National Institute on Aging, National Institutes of Health, Bethesda, MD, 20892, USA; 2 Department of Environmental and Occupational Health Science, University of Washington, Box 357234, 1705 Pacific St, Seattle, WA, 98195, USA

**Keywords:** Arachidonic acid, Docosahexaenoic acid, BDNF, Brain, Polyunsaturated fatty acids (PUFA), Metabolic syndrome, Drebrin, Sucrose, Insulin resistance

## Abstract

**Background:**

In animal models, the metabolic syndrome elicits a cerebral response characterized by altered phospholipid and unesterified fatty acid concentrations and increases in pro-apoptotic inflammatory mediators that may cause synaptic loss and cognitive impairment. We hypothesized that these changes are associated with phospholipase (PLA_2_) enzymes that regulate arachidonic (AA, 20:4n-6) and docosahexaenoic (DHA, 22:6n-6) acid metabolism, major polyunsaturated fatty acids in brain. Male Wistar rats were fed a control or high-sucrose diet for 8 weeks. Brains were assayed for markers of AA metabolism (calcium-dependent cytosolic cPLA_2_ IVA and cyclooxygenases), DHA metabolism (calcium-independent iPLA_2_ VIA and lipoxygenases), brain-derived neurotrophic factor (BDNF), and synaptic integrity (drebrin and synaptophysin). Lipid concentrations were measured in brains subjected to high-energy microwave fixation.

**Results:**

The high-sucrose compared with control diet induced insulin resistance, and increased phosphorylated-cPLA_2_ protein, cPLA_2_ and iPLA_2_ activity and 12-lipoxygenase mRNA, but decreased BDNF mRNA and protein, and drebrin mRNA. The concentration of several n-6 fatty acids in ethanolamine glycerophospholipids and lysophosphatidylcholine was increased, as was unesterified AA concentration. Eicosanoid concentrations (prostaglandin E_2_, thromboxane B_2_ and leukotriene B_4_) did not change.

**Conclusion:**

These findings show upregulated brain AA and DHA metabolism and reduced BDNF and drebrin, but no changes in eicosanoids, in an animal model of the metabolic syndrome. These changes might contribute to altered synaptic plasticity and cognitive impairment in rats and humans with the metabolic syndrome.

## Background

The metabolic syndrome is a clinical disorder characterized by obesity, hypertension, dyslipidemia, glucose intolerance and peripheral inflammation [1-3], and is a risk factor for cognitive decline and mood disorders [4-8]. In rodent models of the metabolic syndrome, behavioral abnormalities have been linked to cerebral hypoglycemia [9] and increased cytokine production [10], and changes in brain lipid metabolism [11,12].

The brain is highly enriched with the polyunsaturated fatty acids (PUFAs), arachidonic acid (AA, 20:4n-6) and docosahexaenoic acid (DHA, 22:6n-3) [13-15], which mostly are esterified in the stereospecifically numbered-2 position of membrane phospholipids. AA and DHA are essential for mediating neuroreceptor signaling, while excessive AA is released during neuroinflammation and excitotoxicity [16-19]. Stimulation of AA signaling by glutamatergic, serotonergic, cholinergic or dopaminergic neuroreceptors, among others, triggers AA release by AA-selective Ca^2+^-dependent cytosolic phospholipase A_2_ (cPLA_2_ IVA) (reviewed in [19]). Unesterified AA is a precursor of prostaglandins, thromboxanes, leukotrienes, and related compounds that have important roles in regulating the brain’s neuroinflammatory response [13-15,20-25]. Stimulation of DHA release from membrane phospholipid *via* DHA-selective calcium-independent iPLA_2_ type VIA is thought to be neuroprotective, and shows anti-inflammatory effects based on *in vitro* and *in vivo* studies [17,26-30]. Disturbed brain AA and DHA metabolism has been linked to a number of neurodegenerative diseases, including Alzheimer’s disease and bipolar disorder [31-33], which are more common in individuals with the metabolic syndrome [4-8].

Brain lipid metabolism is altered in the metabolic syndrome. In a rat model of intracerebroventricular streptozotocin-induced brain insulin resistance and hypoglycemia, cerebral cortex concentrations of ethanolamine glycerophospholipid (EtnGpl) and phosphatidylserine (PtdSer) were decreased, while concentrations of unesterified palmitate, stearate and AA were increased, suggesting increased PLA_2_-mediated membrane degradation [11,12]. Brain phospholipid concentration was reported reduced in a genetic mouse model of diabetes [34]. An increased hippocampal malonaldehyde concentration, a marker of PUFA oxidative degradation, was reported in the hippocampus of genetically obese and hypertensive rats [35]. Taken together, these studies suggest an effect of the metabolic syndrome on the enzymes that regulate brain PUFA metabolism, such as AA-selective cPLA_2_ IVA and iPLA_2_ VIA, which prefers DHA but also can release AA [36,37].

Unesterified AA can be converted to pro-inflammatory and pro-apoptotic secondary mediators, such as prostaglandin E2 (PGE2), thromboxane B2 (TXB2) 82 and leukotriene B4 (LXB4), *via* cyclooxygenase-2 (COX-2) or 5, 12 and 15 lipoxygenase (LOX) [17,38]. These eicosanoids can cause synaptic-dendritic injury by reducing brain levels of trophic factors, such as brain-derived neurotrophic factor (BDNF) [39,40]. In this regard, studies reported decreased BDNF and synaptic loss [41-43] in association with cognitive impairment and behavioral changes, in animal models of the metabolic syndrome [10,41,44]. Although iPLA_2_ VIA can regulate peripheral glucose-stimulated insulin secretion, apoptosis and mitochondrial fatty acid oxidation [45,46], its involvement in modulating brain lipid metabolism in the metabolic syndrome is not known [38].

In view of the reported changes in brain concentrations of phospholipids and PUFAs, and of neuronal loss in animal models of the metabolic syndrome [41-43], we hypothesized correlated disturbances in brain cPLA_2_ IVA and iPLA_2_ VIA expression, fatty acid concentrations, synaptic loss, BDNF, and PGE_2_, TXB_2_ and LXB_4_ concentrations. Such changes have been reported in animal models of neuroinflammation [47-49].

To test this hypothesis, we induced early-stage metabolic syndrome by feeding rats a high-sucrose diet for 8 weeks. In this model, feeding a high-sucrose diet induces time-dependent changes in insulin-resistance and in other markers of the metabolic syndrome after 8 weeks [50], without causing diabetic pathology, fatty liver or weight gain [50-52], which may independently alter brain lipid metabolism [53]. Insulin resistance can be induced in this model without changing the fat composition of the diet, thereby eliminating confounding effects of diet on brain fatty acid composition [53]. In sucrose and control diet fed rats maintained for 8 weeks, we examined brain 1) expression of enzymes involved in AA and DHA metabolism (i.e., mRNA, protein and / or activity of cPLA_2_, iPLA_2_ COX-1, COX-2 and 5-, 12- and 15-LOX); 2) concentrations of PGE_2_, TXB_2_ and LXB_4_; 3) mRNA levels of glial-fibrillary acidic protein (GFAP) and tumor-necrosis factor-α (TNF-α), because of reported changes in these inflammatory markers in animal models of the metabolic syndrome [10]; 4) expression of BDNF, and of synaptophysin and drebrin as markers of synapto-dendritic injury [54]; and 5) esterified fatty acid concentrations in phospholipid subclasses, as well as unesterified fatty acids and lysophosphatidylcholine (lysoPC), as markers of phospholipid degradation. These measurements were performed to test whether phospholipase-mediated phospholipid breakdown occurs in this dietary rat model of the metabolic syndrome, in association with neuroinflammation and synapto-dendritic injury. These pathways are physiologically related because cytokine-induced inflammation, if present in the metabolic syndrome, can alter the expression AA or DHA-selective PLA_2_’s and their downstream metabolites (e.g. eicosanoids) that modulate synapto-dendritic integrity and BDNF expression (reviewed in [55]).

## Results

### Weight gain and food intake

**Figure 1:**
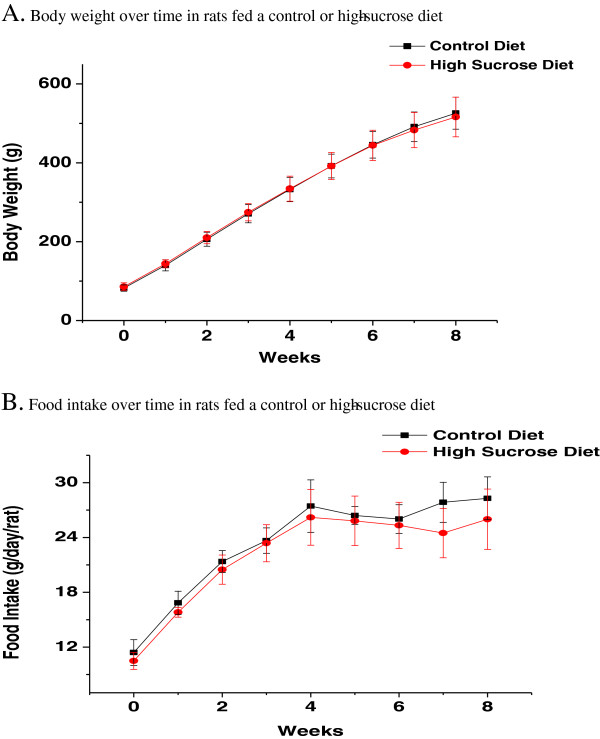
**(A) Body weight and (B) food intake over time in rats fed a control or high- sucrose diet.** Data are means ± SD (n = 6). Two-way repeated measures analysis of variance revealed a significant effect of time only on body weight gain and food intake.

Figure 1 shows body weight (1-A) and food intake (1-B) over the 8-week course of the study. Two-way repeated measures analysis of variance revealed a significant main effect of time on weight and food intake, both of which increased. No significant effect of diet or interaction between diet and time was seen. Consistent with the lack of difference in body weight, weights of brain, liver, adipose tissue, heart, kidneys and testes, collected at the time of sacrifice, did not differ significantly between the two groups (data not shown, p > 0.05 by unpaired *t*-test).

### Oral glucose tolerance test

**Figure 2:**
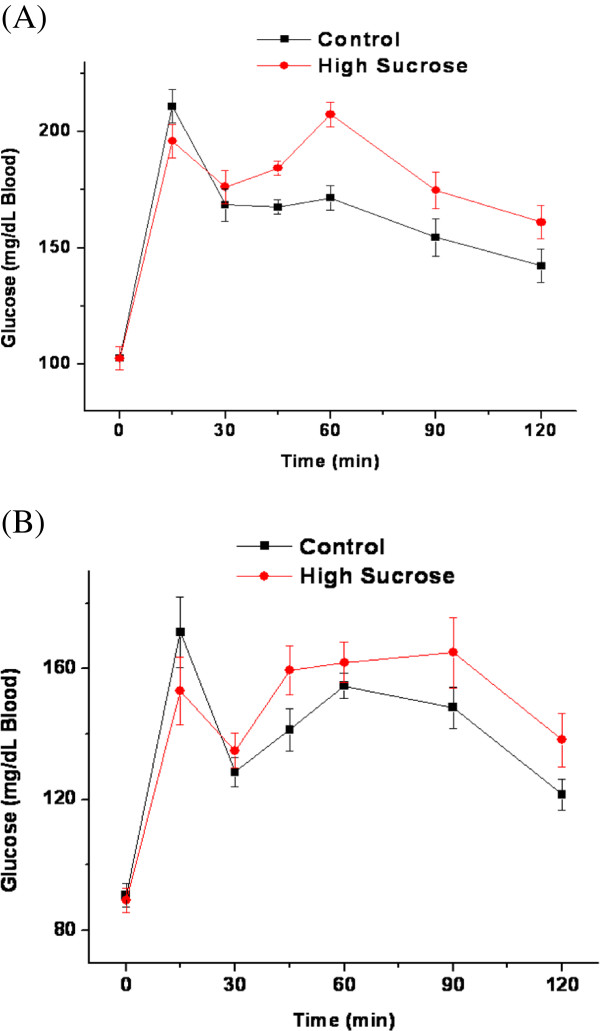
**Oral glucose tolerance test in rats fed a control or high-sucrose diet.** Blood glucose concentrations were measured by a glucometer after a 10 h fast at 0 and 15, 30, 34, 60, 90 and 120 min following gavage with 5g/kg of an oral glucose preload at (**A**) 4 weeks and (**B**) 8 weeks of treatment. Two-way repeated measures analysis of variance showed a significant main effect of diet and time on blood glucose concentrations at 4 and 8 weeks.

Rats fed the high-sucrose diet showed evidence of impaired glucose metabolism, measured by an oral glucose tolerance test at 4 and 8 weeks. Two-way repeated measures analysis of variance showed a significant main effect of diet and time on whole blood glucose concentrations at 4 (Figure 2A) and 8 weeks (Figure 2B). At 4 and 8 weeks, fasting whole blood glucose concentration was similar between the two groups at baseline. The glucose concentration was increased after an oral preload of glucose (5 g/kg) and remained higher than baseline values by the end of the 2 h test. The rise in blood glucose concentration was significantly higher for the high-sucrose compared to control rats, suggesting reduced glucose tolerance in the sucrose- group.

### ß-hydroxybutyrate in whole blood

Fasting ß-hydroxybutyrate concentration in whole blood, a marker of ketosis, was measured at 4 and 8 weeks, before starting the oral glucose tolerance test (Table 1). There was a significant effect of time on fasting ß-hydroxybutyrate concentration, but no effect of treatment or an interaction between time and diet. ß-hydroxybutyrate concentrations were significantly reduced for both dietary groups at 8 weeks compared to 4 weeks (p< 0.05).

**Table 1:** Plasma glucose, insulin and triglyceride concentrations, calculated HOMA index and whole blood ß-hydroxybutyrate concentrations at 4 and 8 weeks of feeding control or high-sucrose diet

	**4 weeks**		**8 weeks**				
**Parameter**	**Control**	**High-sucrose**	**Control**	**High-sucrose**	**Time**	**Diet**	**Time x Diet**
Glucose (mmol/L)	4.0 ± 0.41	4.4 ± 0.1	4.1 ± 1.0	6.0 ± 1.8***	<0.05	<0.01	
Insulin (ng/ml)	0.6 ± 0.4	0.3 ± 0.1	0.7 ± 0.3	1.8 ± 0.9**	<0.001		<0.05
HOMA1 index	2.3 ±1.91	1.4 ± 0.41	4.1 ± 4.1	10.5 ± 4.8**	<0.001	<0.05	<0.05
Triglycerides (mg/ml)	0.9 ± 0.2	0.7 ± 0.2	0.7 ± 0.3	1.5 ± 0.5**	<0.05	<0.05	<0.01
β-hydroxybutyrate (mmol/L)	0.9 ± 0.2	0.9 ± 0.2	0.6 ± 0.2	0.7 ± 0.11	<0.05		

Values are means ± SD (n = 8) ^1^n=7 due to insufficient sample. ^1^n=7 due to insufficient sample. Data were analyzed by two-way repeated measures ANOVA, followed by a one-way ANOVA and Bonferroni’s post-hoc test comparing high-sucrose versus controls at 4 and 8 weeks. **p < 0.01; ***p < 0.001 indicates significant difference compared to 8-week control mean.

### Plasma insulin, glucose and triglyceride concentrations

Rats on the high-sucrose diet showed insulin resistance and hypertriglyceridemia at 8 but not 4 weeks (Table 1). There was a significant interaction between time and diet (p < 0.05) for fasting plasma, insulin, and triglyceride concentrations, and the homeostasis model assessment (HOMA) insulin-resistance index, calculated as previously described [56]. There was a significant main effect of diet on plasma glucose and triglyceride concentrations, and on the HOMA index. Time was a significant factor affecting glucose, insulin and triglyceride concentrations, and the HOMA index. Compared to controls, rats fed the high-sucrose diet had significantly higher plasma concentrations of glucose and triglycerides and HOMA index at 8 weeks.

### cPLA_2_ mRNA and protein

**Figure 3:**
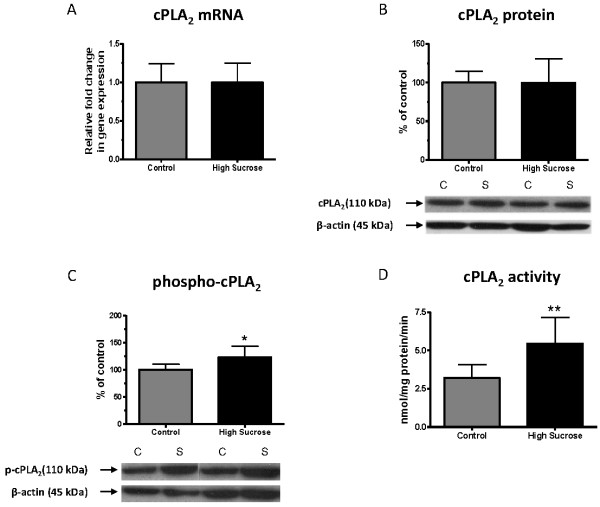
**mRNA, protein and activity of cPLA**_**2**_**and phosphorylated (phospho)-cPLA**_**2**_**protein in brain of rats fed the control or high-sucrose diet for 8 weeks.A**) cPLA_2_ (IVA) mRNA, **B**) cPLA_2_ (IVA) protein, **C**) phospho- cPLA_2_ (IVA) protein and **D**) cPLA_2_ activity. Data are expressed as the relative level of the cPLA_2_ normalized to the endogenous control (β-globulin) using the ΔΔ*C*_*T*_ method. Protein level is the ratio of optical density of cPLA_2_ to β–actin, expressed as percent of control. Values are mean ± SD (n = 8 for both groups). *p < 0.05, **p < 0.01 by unpaired *t*-test.

mRNA and protein levels of brain cPLA_2_ IVA did not differ significantly between rats fed the high sucrose and the control diet for 8 weeks (Figure 3A and 3B). The protein level of phosphorylated-cPLA_2_ (phospho-cPLA_2_), which represents the active form of cPLA_2_[57,58], was increased significantly in the high-sucrose group compared to the control group (Figure 3C). Consistent with the increase, in phosphorylated-cPLA_2_ activity of cPLA_2_ was significantly increased in the high-sucrose group (Figure 3D).

### iPLA_2_ and sPLA_2_ mRNA, protein and activity

**Figure 4:**
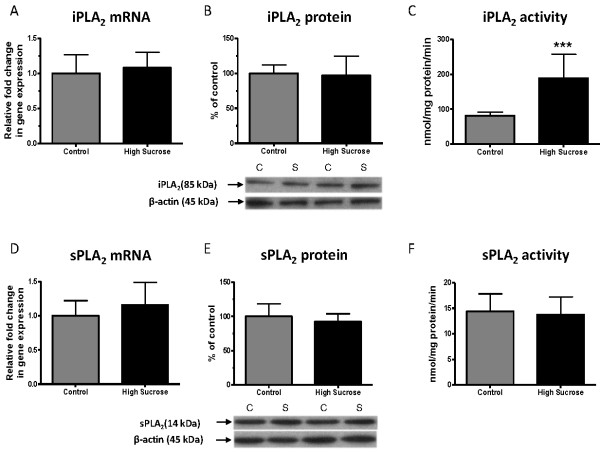
**mRNA, protein and activity of brain iPLA**_**2**_**and sPLA**_**2**_**in rats fed the control or high-sucrose diet for 8 weeks.** iPLA_2_ (VIA) **A**) mRNA, **B**) protein and **C**) activity; sPLA_2_ (IIA) **E**) mRNA, **F**) protein and G) activity. Data are expressed as the relative level of the iPLA_2_ and sPLA_2_ normalized to the endogenous control (β-globulin) using the ΔΔ*C*_*T*_ method. Protein level is the ratio of optical density of iPLA_2_ and sPLA_2_ to β–actin, expressed as percent of control. Values are mean ± SD (n = 8 for both groups). ***p < 0.001 by unpaired *t*-test.

iPLA_2_ VIA and sPLA_2_ IIA mRNA and protein did not differ significantly between rats fed the high sucrose and the control diet for 8 weeks (Figure 4A, B, D, E). The activity of iPLA_2_ was significantly increased in the high-sucrose group (Figure 4C), whereas sPLA_2_ activity did not differ significantly between the groups (Figure 4F).

### PGE_2_, TXB_2_ and LTB_4_ concentrations

**Figure 5:**
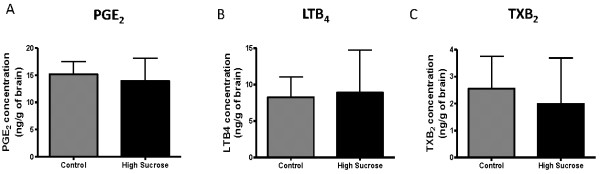
**Levels of A) PGE**_**2**_**, B) LTB**_**4**_**, and C) TXB**_**2**_**in brain of rats fed a control or high-sucrose diet for 8 weeks.** PGE_2_, LTB_4_, and TXB_2_ were extracted according to the method of Radin [82] and analyzed using a polyclonal enzyme-linked immunosorbent assay. Values are mean ± SD (n = 8 for both groups). *p < 0.05 by unpaired *t*-test.

There was no significant difference in PGE_2_, LTB_4_ or TXB_2_ concentration between the two groups (Figure 5A-C).

### GFAP, TNF-α, COX and LOX mRNA

There was no significant change in GFAP or TNF-α mRNA (data not shown). mRNA levels of COX-1 and COX-2 also were not affected by the high-sucrose diet (data not shown), nor was 5- or 15-LOX mRNA changed significantly. Brain 12-LOX mRNA was increased significantly (control, 1.00 ± 0.07; high-sucrose, 1.22 ± 0.05; p < 0.05 by unpaired t-test).

### mRNA and protein levels of BDNF and synaptic markers

**Figure 6:**
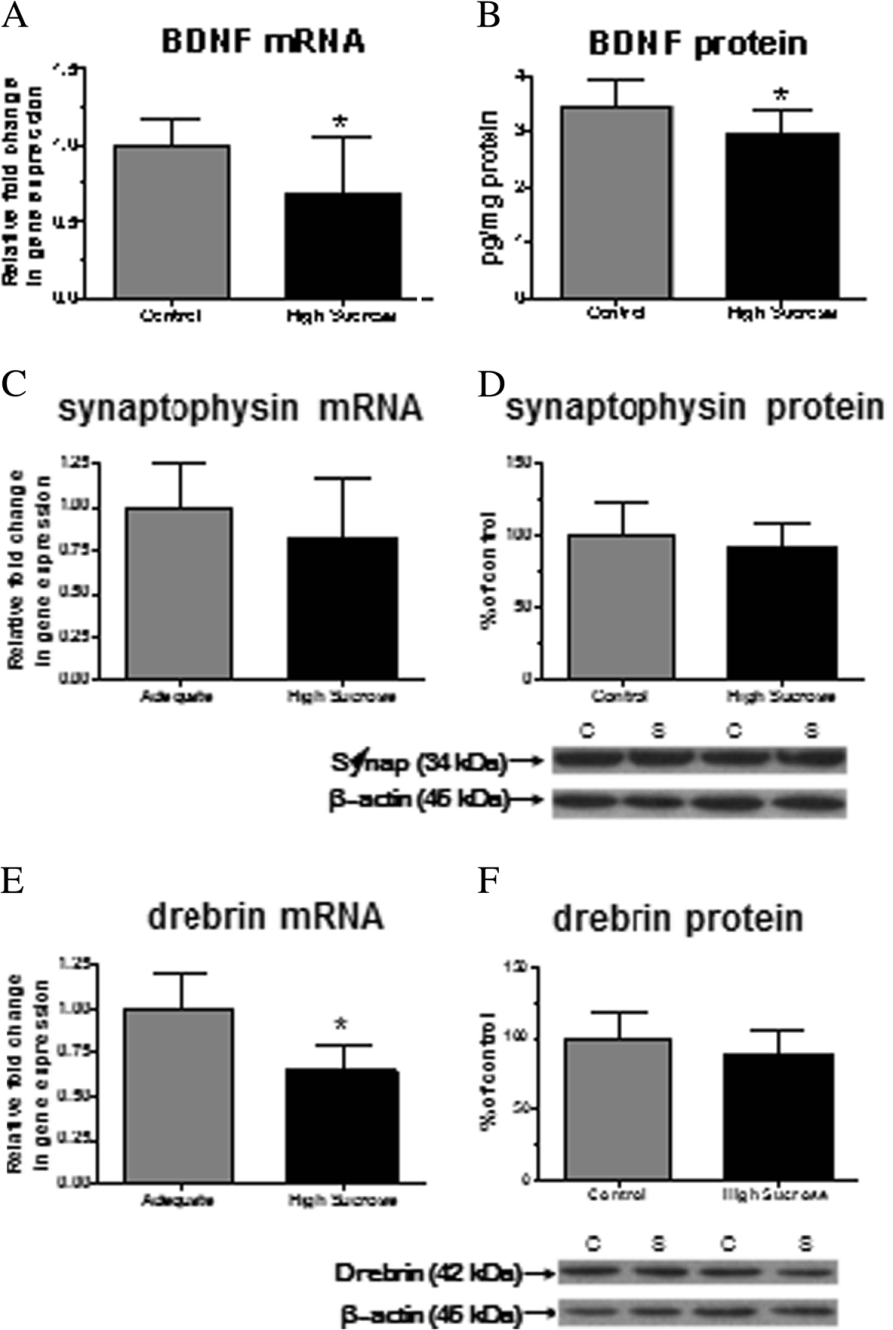
**mRNA and protein levels of BDNF and synaptic markers in brain of rats fed a control or high-sucrose diet for 8 weeks.A**) BDNF mRNA, **B**) BDNF protein, **C**) synaptophysin mRNA, **D**) synaptophysin protein, **E**) drebrin mRNA and **F**) drebrin protein. Data are expressed as relative level of BDNF, synaptophysin and drebrin normalized to endogenous control (β-globulin) using the ΔΔ*C*_*T*_ method. The BDNF protein level was measured by an ELISA kit. The protein level is the ratio of optical density of synaptophysin or drebrin to β–actin, expressed as percent of control. Values are mean ± SD (n = 8 for both groups). *p < 0.05 by unpaired *t*-test.

Compared with control diet, the high-sucrose diet decreased significantly mean protein and mRNA levels of BDNF (Figure 6A and B). mRNA and protein levels of the pre-synaptic marker synaptophysin did not differ significantly between the groups (Figure 6C and D). There was a significant decrease in mRNA of the post-synaptic dendritic spine marker [54], drebrin (Figure 6E), but no significant change in its protein level (Figure 6F).

### Brain fatty acid concentrations

**Figure 7:**
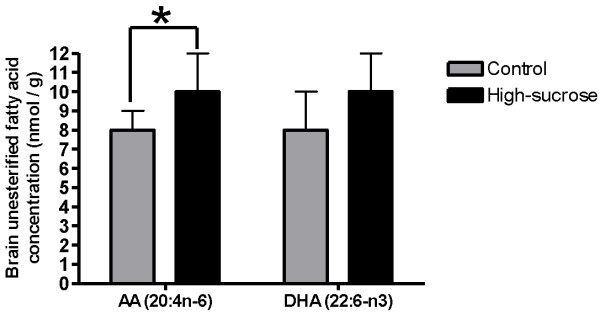
**Brain concentrations of unesterified AA and DHA in rats fed a control or high-sucrose diet for 8 weeks.** Values are mean ± SD (n = 8 for both groups). *p < 0.05 by unpaired *t*-test.

There were few significant differences in brain esterified fatty concentrations (data not shown): a 12-16% increase in AA (6262 ± 459 nmol/g brain wet wt, control, 7234 ± 468 nmol/g wet wt, sucrose) and adrenate (22:4n-6; 3321 ± 275 nmol/g wet wt, control, 3738 ± 158 nmol/g wet wt, sucrose) in EtnGpl, and a 29% increase in dihomo-γ-linolenic acid (20:3n-6; 7 ± 1 nmol/g wt wet, control, 9 ± 1 nmol/g wt wet, sucrose) in lysoPC in rats on the high-sucrose compared to control diet (p < 0.05). For unesterified fatty acids, one sample from the high-sucrose group was excluded from the analysis because its unesterified AA concentration was 3-fold higher than the mean, which suggests ischemia caused by incomplete microwave-fixation [59]. The unesterified AA concentration was significantly increased (by 20%) in the high-sucrose diet group, whereas the DHA concentration did not differ significantly from control (Figure 7). Other unesterified fatty acid concentrations also did not differ between the groups (data not shown).

## Discussion

Rats fed the high-sucrose diet (60% sucrose) for 8 weeks did not show obesity or increased food intake, but developed hypertriglyceridemia and insulin resistance, two components of the metabolic syndrome, as reported [50-52]. At 8 weeks, some brain enzymatic markers of AA and DHA metabolism were increased significantly in the high sucrose compared with control diet group (e.g., protein level of phospho-cPLA_2_, activities of cPLA_2_ and iPLA_2_) as was the esterified AA concentration in EtnGpl and unesterified AA concentration. BDNF mRNA and protein and drebrin mRNA were reduced, but synaptophysin mRNA and protein were not altered. Feeding the high-sucrose diet for 8 weeks did not change PGE_2_, TXB_2_ or LTB_4_ concentration significantly. Because the 8-week high-sucrose feeding paradigm represents early-stage metabolic syndrome in the absence of pathological diabetes or obesity [50,51], these findings demonstrate changes in brain PUFA metabolizing enzymes and composition in association with reduced BDNF and drebrin mRNA at an early disease stage.

The upregulation of brain cPLA_2_ and iPLA_2_ enzyme activities (Figure 3 and 4) in the high-sucrose fed rats suggests an increase in brain AA and DHA metabolism. In this regard, disturbed saturated brain fatty acid metabolism has been reported in humans and rats with the metabolic syndrome [60,61]. A positron emission tomography study demonstrated increased brain uptake of ^11^C]palmitate and ^18^F]fluoro-6-thia-heptadecanoic acid in patients with the metabolic syndrome [60]. Hypothalamic concentrations of long-chain saturated acyl-CoAs were increased in a high-fat diet animal model of the metabolic syndrome, also indicating increased metabolism of long-chain saturated fatty acids [61]. Taken together, the results suggest non-specific upregulation in brain fatty acid metabolism, including PUFAs, associated with the metabolic syndrome. Upregulated AA or DHA metabolism could be directly confirmed in this animal model, using quantitative autoradiography to image fatty acid uptake following radiotracer injection, or can be examined in humans using positron emission tomography [31,38,62].

The released fatty acids may be alternative energy substrates to glucose for brain metabolism, due to cerebral hypoglycemia caused by insulin-resistance. This is consistent with evidence of increased brain activity of carnitine palmitoyltransferase (which regulates fatty acid entry from the acyl-CoA pool into mitochondria for later β-oxidation) in an animal model of the metabolic syndrome [34]. ^14^C-palmitate conversion to ^14^C-CO_2_ also was increased in mitochondrial brain extracts of diabetic (db/db) mice [34].

Brain cPLA_2_ activity and phospho-cPLA_2_ protein, a marker of activated cPLA_2_[57,58], were increased in the high-sucrose fed rats in the absence of changes in cPLA_2_ mRNA or protein, suggesting post-translational modification and upregulated brain AA metabolism, consistent with the increased unesterified AA concentration (Figure 7). Increased activation of cPLA_2_ may reflect excitotoxicity associated with increased influx of extracellular calcium into the cell via ionotropic glutamatergic receptors [63]. Since cPLA_2_ also is functionally coupled via G-proteins to dopaminergic, serotonergic and muscarinic neuroreceptors [19], an increase in its activity suggests disturbed G-protein neuroreceptor signaling in the metabolic syndrome [64]. Cytokine receptor activation may also initiate cPLA_2_ activation [65], although our findings do not suggest an increase in cytokine expression in rats fed the high-sucrose diet.

iPLA_2_ is insensitive to extracellular calcium influx into the neuron [24,63], but can be activated by intracellular calcium (at mM concentrations) released from the endoplasmic reticulum by the calcium-releasing ryanodine receptor [66]. Mobilization of intracellular calcium stores can be mediated by increased intracellular unesterified AA levels, which was reported to activate the ryanodine receptor *in vitro*[66]. This is in agreement with the finding that the unesterified AA concentration was increased in the high-sucrose diet rats (Figure 7). Likely, this increase in AA concentration occurred intracellularly, since sPLA_2_, which releases AA extracellularly, was not changed significantly (Figure 4).

Concentrations of pro-inflammatory eicosanoids (PGE_2_, TXB_2_ and LTB_4_) did not differ between the groups (Figure 5). It is possible, however, that changes in eicosanoids or cytokines [65] occurred in specific brain regions such as the hippocampus, as reported in genetically diabetic mice, or that longer administration of the high-sucrose diet sufficient to initiate diabetes would increase whole brain cytokine levels [10]. However, consistent with the lack of significant changes in the three eicosanoids, we did not find significant changes in mRNA levels for COX-1, COX-2, 5- or 15-LOX in the high-sucrose fed rats, nor in TNF-α or GFAP mRNA, suggesting the absence of neuroinflammation, since transcription of these molecular markers occurs within transcriptional circuits related to neuroinflammation [67-69].

Whole brain BDNF mRNA and protein levels were reduced in the high-sucrose group (Figure 6), in agreement with previous studies that showed reduced BDNF levels in animal models of the metabolic syndrome with behavioral impairment [10,41,42]. Reduced BDNF expression was not mediated by pro-inflammatory eicosanoids, which were not changed. One possibility is that the increased unesterified AA concentration in the high-sucrose animals decreased BDNF and induced apoptosis, as reported in cultured spinal cord neurons [70]. Reduced BDNF expression in the sucrose-fed rats may have promoted dendritic injury, which was indirectly suggested by the reduction in drebrin mRNA (Figure 6), or have altered the cellular dynamics and structural organization of dendritic spines in the absence of changes in drebrin protein. Changes in dendritic morphology and dynamics could be the topic of future studies. Additionally, more severe changes in synaptic structure are likely to occur with prolonged exposure to the high-sucrose diet, since the 8-week feeding paradigm causes only early-stage metabolic syndrome without obesity, diabetes or liver damage [41,44,52].

Contrary to reports using other models involving central insulin resistance [12,34,42], we did not find evidence of phospholipid degradation in the brain, since phospholipid mass, derived by the summation of total fatty acids within each phospholipid class, did not differ between the dietary groups (Table 1). Also, lysoPC, a marker of phospholipid breakdown, was not changed (Table 1). The changes in phospholipid fatty acid concentrations were relatively minor, and were significant only for a few n-6 PUFAs in EtnGpl (AA and 22:4n-6) and in lysoPC (20:3n-6).

## Conclusion

In summary, brain enzymatic markers of AA and DHA metabolism were increased in a rat model of early-stage metabolic syndrome, in association with reduced BDNF mRNA and protein, and drebrin mRNA. Increases in cPLA_2_ and iPLA_2_ activities support the notion of phospholipase-mediated neurodegeneration [11,12,34,35]. The decreases in BDNF and drebrin suggest increased susceptibility to synapto-dendritic injury.

In the future, an upregulation in brain AA and DHA metabolism associated with the metabolic syndrome might be imaged in humans with positron-emitting tomography using radiolabeled AA or DHA [31,62], as biomarkers of disease progression [4,5,71]. Therapeutic strategies aimed at downregulating brain PUFA metabolism, such as the administration of carnitine palmitoyltransferase inhibitors [72,73] or PLA_2_ inhibitors (e.g. the mood stabilizers, lithium and carbamazepine), might be effective in slowing the progression of brain lipid abnormalities identified in this study, the associated changes in synaptic loss and possibly, cognitive dysfunction in the metabolic syndrome.

## Methods

### Animals

The protocol was approved by the Animal Care and Use Committee of the *Eunice Kennedy Schriver* National Institute of Child Health and Human Development and followed the National Institutes of Health Guide for the Care and Use of Laboratory Animals (NIH Publication No. 80–23). Post-weaning male Wistar rats purchased from Charles River Laboratories (Portage, MI, USA) were housed in an animal facility with regulated temperature, humidity, and a 12 h light/12 h dark cycle. After weaning, pups were divided randomly into control diet (n = 14) and high sucrose diet (n = 14) groups. The metabolic syndrome was confirmed in 8 out of 14 rats per dietary group, by measuring body weight and food intake weekly, and measuring tail vein plasma glucose, insulin and triglyceride concentrations at 4 and 8 weeks. An oral glucose tolerance test was also administered at 4 and 8 weeks to the same rats (n = 8 rats per group) as described below. After 8 weeks on a chosen diet, half the rats from each dietary group (n = 8 per group) were asphyxiated by CO_2_ inhalation, decapitated and their brains excised rapidly, frozen in 2-methylbutane with dry ice at −50°C, and stored at −80°C until use. Brain, testes, adipose tissue, liver and heart were collected, weighed, frozen in 2-methylbutane and stored at −80°C.

Brain lipids and eicosanoids (PGE_2_, TXB_2_ and LTB_4_) were measured in the remaining animals (n = 6 per roup) that had undergone a catheter implantation surgery followed by a 2-h infusion protocol of [D^5^-α-linolenic acid (17, 17, 18, 18, 18-D^5^) and [U-^13^C]-linoleic acid (Spectra Stable Isotopes, Columbia, MD, USA) to assess liver PUFA kinetics (Taha et al., unpublished). After the 2 h infusion, the rats were lightly anesthetized with sodium pentobarbital (50 mg/kg; Abbott Laboratories, Chicago, IL, USA) and subjected to head-focused microwave irradiation stop brain lipid metabolism (5.5 kW, 4.8 s; Cober Electronics, Stamford, CT, USA) [74]. Brains were excised, separated sagittally into two halves and stored at −80°C until analyzed.

### Diets

The control and high sucrose diets were obtained from Dyets Inc. (Bethlehem, PA, USA), and were based on the AIN-93G formulation [75]. The diets were isocaloric and identical in macronutrient and micronutrient composition, but differed in carbohydrate composition. The control diet contained cornstarch (150 g/kg diet), sucrose (100 g/kg), dextrose (200 g/Kg) and maltose dextrin (150 g/kg). The high-sucrose diet contained sucrose (600 g/kg) as the sole carbohydrate source (Table 2). The fatty acid composition of the diets was identical, and contained 7.8 μmol/g α-LNA (4.6% total fatty acid), which is the minimum level of α-LNA for n−3 PUFA adequacy in rodents, 40 μmol/g LA (25% total fatty acid), 110 μmol/g saturated fatty acid (68.5% of total), and 10 μmol/g monounsaturated fatty acid [76]. Other PUFAs, including AA and DHA were absent.

**Table 2:** Composition of control diet and high-sucrose diet

**Ingredients**	**Control diet**	**High-sucrose diet**
	*(gram/Kg diet)*	
Protein	200	200
Casein	200	200
Cornstarch	150	0
Sucrose	100	600
Dextrose	200	0
Maltose Dextrin	150	0
Hydrogenated coconut oil	60	60
Safflower oil	32.3	32.3
Flaxseed oil	77	77
Cellulose	50	50
Salts	35	35
Vitamins	10	10
L-Cystine	3	3
Choline bitartrate	2.5	2.5
t-Butylhydroquinone	0.02	0.02

### Plasma glucose, insulin, and triglyceride measurement

Blood was collected at 4 and 8 weeks from the tail vein after an overnight 10 h fast (n = 8 per group), and plasma glucose, insulin and triglyceride concentrations were determined with a glucose oxidase kit (Sigma), an Insulin ELISA kit (Alpco Diagnostics, Salem, NH, USA) and a triglyceride kit (Sigma), respectively. The insulin and glucose concentrations were used to calculate the ‘homeostasis model assessment’ (HOMA) index of insulin resistance, by multiplying glucose (mmol/L) and insulin (mU/L) concentrations, and dividing by 22.5 [56].

### Oral glucose tolerance test and β-hydroxybutyrate measurement

An oral glucose tolerance test [77,78] was performed at 4 and 8 weeks post-weaning, 2 days after the tail vein blood withdrawals described above. For this test, blood was obtained by tail-prick using a sharp needle. After an overnight 10 h fast, baseline blood glucose and ß-hydroxybutyrate concentrations were assayed using a commercial glucometer (LifeScan, Milpitas, CA, USA). The rats then were gavaged with 0.375 g glucose/ml (5 g glucose/kg body wt), and blood glucose concentrations were determined 15, 30, 45, 60, 90, and 120 min later.

### Brain total fatty acid concentration

Brain total lipids were extracted by the Folch method [79]. An aliquot of the total lipid extract was methylated with 1% H_2_SO_4_-methanol for 3 h at 70°C, or separated into phospholipid subfractions with thin layer chromatography (TLC) using heptane / diethyl ether / acetic acid (60:40:3 v/v/v) as a solvent. Unesterified fatty acids were separated with TLC using chloroform / methanol / acetic acid / water (60:50:1:4 v/v/v/v). Prior to methylation, di-17:0 PC was added as an internal standard to total lipids and phospholipid subfractions. Unesterified 17:0 was added as an internal standard to unesterified fatty acids. Samples were methylated with 1% H_2_SO_4_-methanol for 3 h at 70°C. The resulting fatty acid methyl esters were extracted and analyzed using a gas chromatograph (6890N, Agilent Technologies, Palo Alto, CA, USA) equipped with an SP-2330 fused silica capillary column (30 m×0.25 mm i.d., 0.25 μm film thickness) (Supelco, Bellefonte, PA, USA) and a flame ionization detector. Concentrations were calculated by proportional comparison of peak areas to the area of the 17:0 internal standard.

### Preparation of cytoplasmic and membrane extracts

Cytoplasmic and membrane extracts for Western blots were prepared using a compartmental protein extraction kit according to the manufacturer’s instructions (Millipore, Temecula, CA, USA). Protein concentrations of cytoplasmic and membrane extracts were determined using Bio-Rad Protein Reagent (Bio-Rad, Hercules, CA, USA).

### Western Blot Analysis

Proteins from cytoplasmic (50 μg) and membrane extracts (50 μg) were separated on 4-20% SDS-polyacrylamide gels (PAGE) (Bio-Rad). Following SDS-PAGE, the proteins were electrophoretically transferred to a nitrocellulose membrane. Protein blots were incubated overnight at 4°C in Tris-buffered saline (TBS) buffer, containing 5% nonfat dried milk and 0.1% Tween-20, with specific primary antibodies (1:1000 dilution) for the group IVA cPLA_2_, phospho-cPLA_2_, group IIA secretory sPLA_2_, group VIA iPLA_2_ (Santa Cruz Biotech, Santa Cruz, CA), drebrin, synaptophysin (Cell Signaling, Beverly, MA), and β-actin (Sigma-Aldrich, St. Louis, MO). Protein blots were incubated with appropriate HRP-conjugated secondary antibodies (Cell Signaling) and visualized using a chemiluminescence reaction (Amersham, Piscataway, NJ) on X-ray film (XAR-5, Kodak, Rochester, NY). Optical densities of immunoblot bands were measured using Alpha Innotech Software (Alpha Innotech, San Leandro, CA) and were normalized to β-actin to correct for unequal loading. All experiments were carried out three times with 8 independent samples per group. Values are expressed as percent of control.

### BDNF protein levels

BDNF protein levels were measured in brain cytosolic extracts using an ELISA kit. according to the manufacturer’s instructions (Millipore, Temecula, CA). Values are expressed in pmol/mg protein.

### Total RNA isolation and real time RT-PCR

Total RNA was prepared from brain using commercial kits (RNeasy Lipid Tissue Kit; Qiagen, Valencia, CA). cDNA was prepared from total RNA using a high-capacity cDNA Archive Kit (Applied Biosystems, Foster City, CA). mRNA levels were measured by real time quantitative RT-PCR, using the ABI PRISM 7000 sequence detection system (Applied Biosystems). For specific primers and probes for target genes, TaqMan^R^ gene expression assays, purchased from Applied Biosystems, consisted of a 20X mix of unlabeled PCR primers and Taqman minor groove binder probe (FAM dye-labeled). The fold change in gene expression was determined using the ΔΔC_T_ method [80]. Data are expressed as the relative level of the target gene in the high-sucrose animals normalized to the endogenous control (β-globulin) and relative to the control rats (calibrator). All experiments were carried out in duplicates with 8 independent samples per group.

### Phospholipase A_2_ activities

#### Sample preparation

Brain tissue was homogenized with 3 vol of homogenization buffer (10 mM HEPES, pH 7.5, containing 1 mM EDTA, 0.34 μM sucrose and protease inhibitor cocktail (Roche, Indianapolis, IN)), using a glass homogenizer. The homogenized sample was centrifuged at 100,000 g for 1 h at 4°C, and the supernatant was used for all PLA_2_ enzyme activity analyses. Supernatants were kept at −80°C until use. The protein concentration was analyzed by the Bradford assay (Bio-Rad) [81].

#### Enzyme assay with radioisotope method

The final incubation volume was 0.5 ml. To measure cPLA_2_ activity, the cytosolic fraction (0.3 mg protein in one assay, ~50 μl) was mixed with 100 mM HEPES, pH 7.5 containing 80 μM Ca^2+^, 2 mM dithiothreitol and 0.1 mg/ml fatty acid-free bovine serum albumin (total volume = 450 μl). The enzyme reaction was started by adding fifty μl of substrate solution containing 100 μM 1-palmitoyl-2-arachidonoyl-*sn*-glycerol-3-phosphorylcholine and phosphatidylinositol 4,5-bisphosphate (97:3) (Avanti Polar Lipids, Alabaster, AL, USA), and approximately 100,000 dpm of 1-palmitoyl-2-[1-^14^C] arachidonoyl-*sn*-glycerol-3-phosphorylcholine (specific activity of 60 mCi/mmol, PerkinElmer, Boston, MA) in 400 μM triton X-100 per assay, To measure iPLA_2_ activity, the cytosolic fraction (0.3 mg protein in one assay) was mixed with 100 mM HEPES, pH 7.5, 5 mM EDTA, 2 mM dithiothreitol, and 1 mM ATP (total volume = 450 μl). Fifty μl substrate mixture of 100 μM 1-palmitoyl-2-palmitoyl-*sn*-glycerol-3-phosphorylcholine containing approximately 100,000 dpm of 1-palmitoyl-2-[1-^14^C] palmitoyl-*sn*-glycerol-3-phosphorylcholine (specific activity of 53 mCi/mmol, Buckinghamshire, UK) in 400 μM Triton X-100 was added to start the enzyme reaction.

#### Substrate preparation for radioisotope method

Substrates for the iPLA_2_ and cPLA_2_ activity analyses described above were prepared daily. Appropriate amounts of cold and radiolabeled phospholipids were added to an appropriate amount of Triton X-100, and the mixture was dried with nitrogen gas. Water was added to the residues to give a 10x lipid mixture (1 mM phospholipid, 1,000,000 dpm, and 4 mM Triton X-100), which was mixed vigorously.

#### Enzyme assay

The cytosolic fraction (0.3 mg in one assay) was mixed with the assay mixture (total volume of 450 μl), and 50 μl substrate mixture was added to start the enzyme reaction. The reaction mixture was incubated for 30 min at 40°C, and then 2.5 ml of Dole reagent (2-propanol, heptane: 0.5M H_2_SO_4_, 400:100:20, vol/vol/vol) was added to stop the reaction. One and a half ml of heptane and 1.5 ml H_2_O were added to the mixture, followed by vortexing and centrifugation at 3000 rpm for 5 minutes. The upper phase (about 2 ml) was transferred to a tube containing 200 mg of silicic acid (200–400 mesh), followed by vortexing and centrifugation. The supernatant (1.5 ml) was transferred to a scintillation vial, and scintillation cocktail was added (Ready Safe™ plus 1% glacial acetic acid). Radioactivity of the released unesterified fatty acid from the phospholipid substrate was counted on a liquid scintillation counter (2200CA, TRI-CARB^®^, Packard Instruments, Meriden, CT, USA). iPLA_2_ and cPLA_2_ activities were expressed as the release rate of fatty acid from phospholipids.

#### sPLA_2_ activity

sPLA_2_ activity was measured using an appropriate assay kit (Cayman, Ann Arbor, MI, USA), according to the manufacturer’s instructions.

### PGE_2_, TXB_2_, and LTB_4_ concentration

PGE_2_, TXB_2_, and LTB_4_ were extracted according to the method of Radin [82]. A portion of the extract was dried under nitrogen and assayed for PGE_2_, TXB_2_, and LTB_4_ using a polyclonal enzyme-linked immunosorbent assay according to the manufacturer’s instructions (Oxford Biomedical Research, Oxford, MI).

### Data and statistics

Data are presented as means ± SD (n = 8 for each group). A two-way repeated measured analysis of variance (ANOVA) was used to test for effects of time and treatment on body weight, food intake, and insulin, glucose, β-hydroxybutyrate and triglyceride concentrations, and the response to an oral glucose preload performed at 4 and 8 weeks. An unpaired Student’s *t*-test was used to compare means, taking p < 0.05 as the cut off for statistical significance.

## Abbreviations

AA: arachidonic acid; ANOVA: analysis of variance; BDNF: brain derived neurotrophic factor; cPLA_2_: cytosolic phospholipase A_2_; DHA: docosahexaenoic acid; EtnGpl: ethanolamine glycerophospholipids; HOMA: homeostasis model assessment; iPLA_2_: calcium-independent PLA_2_; i.c.v.: intracerebroventricular; LXB_4_: leukotriene B_4_; lysoPC: lysophosphatidylcholine; PAGE: polyacrylamide gels; PGE_2_: prostaglandin E_2_; PtdSer: phosphatidylserine; sPLA_2_: secretory PLA_2_; TXB_2_: thromboxane B_2_.

## Competing interests

The authors declare that they have no competing interests.

## Authors’ contributions

AYT, FG and SIR conceived and designed the study. AYT and FG carried out the animal experiments. HWK, AYT, YC, FG and IR were involved in the analysis. AYT, HWK and SIR were involved in writing and editing the manuscript. All authors read and approved the final manuscript.
